# Quality of care in the last year of life: adaptation and validation of the German “Views of Informal Carers’ Evaluation of Services – Last Year of Life – Cologne”

**DOI:** 10.1186/s12913-022-08700-0

**Published:** 2022-11-28

**Authors:** Gloria Dust, Nicolas Schippel, Stephanie Stock, Julia Strupp, Raymond Voltz, Christian Rietz, On behalf of the CoRe-Net Co-applicants

**Affiliations:** 1 Faculty of Medicine and University Hospital, Department of Palliative Medicine, University of Cologne, 50924 Cologne, Germany; 2 Faculty of Medicine and University Hospital, Institute of Health Economics and Clinical Epidemiology, University of Cologne, Cologne, Germany; 3 Faculty of Medicine and University Hospital, Center for Integrated Oncology Aachen, University of Cologne, Bonn Cologne Duesseldorf (CIO ABCD), Cologne, Germany; 4 Faculty of Medicine and University Hospital, Center for Health Services Research, University of Cologne, Cologne, Germany; 5 Faculty of Educational and Social Sciences, Department of Educational Science, University of Education Heidelberg, Heidelberg, Germany

**Keywords:** Quality of care, Validation, Assessment, Proxy, Last year of life, Palliative care

## Abstract

**Background:**

To inform quality improvement and strengthen services provided in the last year of life, measuring quality of care is essential. For Germany, data on care experiences in the last year of life that go beyond diagnoses and care settings are still rare. The aim of this study was to develop and validate a German version of the ‘Views of Informal Carers’ Evaluation of Services – Short Form (VOICES-SF)’ suitable to assess the quality of care and services received across settings and healthcare providers in the German setting in the last year of life (VOICES-LYOL-Cologne).

**Methods:**

VOICES-SF was adapted and translated following the ‘TRAPD’ team approach. Data collected in a retrospective cross-sectional survey with bereaved relatives in the region of Cologne, Germany were used to assess validity and reliability.

**Results:**

Data from 351 bereaved relatives of adult decedents were analysed. The VOICES-LYOL-Cologne demonstrated construct validity in performing according to expected patterns, i.e. correlation of scores to care experiences and significant variability based on care settings. It further correlated with the PACIC-S9 Proxy, indicating good criterion validity. The newly added scale “subjective experiences of process and outcome of care in the last year of life” showed good internal consistency for each given care setting, except for the homecare setting. Test-retest analyses revealed no significant differences in satisfaction ratings according to the length of time since the patient’s death. Overall, our data demonstrated the feasibility of collecting patient care experiences reported by proxy-respondents across multiple care settings.

**Conclusion:**

VOICES-LYOL-Cologne is the first German instrument to analyse care experiences in the last year of life in a comprehensive manner and encourages further research in German-speaking countries. This instrument enables the comparison of quality of care between settings and may be used to inform local and national quality improvement activities.

**Trial registration:**

This study was registered in the German Clinical Trials Register (DRKS00011925; Date of registration: 13/06/2017).

## Background

The last year of life constitutes a particularly emotional and vulnerable period for patients as well as for their relatives. It may be characterized by the experience of physical and mental decline, by symptom burden, and the need of support from others [1]. Especially older adults are confronted with multiple illnesses and higher care and medical service utilisation. Health-care needs are complex and admission to a hospital, nursing home or hospice is common [2, 3]. Health care systems are challenged to respond effectively to the intense needs of these patients [4]. In 2015 Germany adopted a law to improve and extend palliative and hospice care [5]. Depending on their needs, patients may receive generalist palliative care or specialist palliative care provided by health care professionals with expert knowledge, skills and attitudes [6]. Initial studies concluded that available services do not yet meet patient needs, since the provision of palliative care often starts too late or is restricted to cancer patients [7]. Thus, there is a need to systematize approaches to assess performance and quality of care provided in the last year of life.

Measuring the quality of care is a core priority to strengthen these services [8]. To conceptualize quality of care, Donabedian developed a three-part approach by assessing elements of (1) “structure” – the attributes of the settings in which care occurs, (2) “process” – the activities in giving (practitioner) and receiving (patient) care, and (3) “outcome” – the effects of care on the health status of patients and populations [9]. Key aspects to measure and assure quality are patient preferences. User perspectives are particularly important in end-of-life care research because commonly used endpoints, such as morbidity and mortality, are not useful for this patient population [10]. Recently, there has been increased development and use of patient reported experience measures (PREM) in order to obtain this information. PREM assess the way in which patients experience the process of care including satisfaction (e.g. satisfaction with information given), subjective experiences (e.g. pain control), objective experiences (e.g. waiting time) and observations of healthcare providers’ behaviour (e.g. whether or not a patient was given discharge information by a nurse). PREM allow patients to provide direct feedback on their care to drive improvement in services [11].

Yet, evaluating care experiences at the end of life presents unique challenges, as many patients are too ill to participate or are not identified as dying. Bereaved relatives are therefore often used as proxy to investigate patients’ views about their care [12]. In addition, care in the last year of life comprises a wide range of services important to patients, which requires a multidimensional assessment approach. Furthermore, measures need to enable the respondent to differentiate between care received by different providers. Several instruments have been developed to measure patient care experience comprising a wide variation in content areas. Many focus on the dying phase or the last weeks to months of life, or they are limited to a single care setting [4]. There is one survey, the “Views of Informal Carers – Evaluation of Services (VOICES)” from England that assesses patient and relative experiences of care across different settings and providers in the last three months of life [13]. A short form, VOICESSF, was developed following extensive research and consultation with patients, relatives and healthcare professionals to be applied in the national representative survey in England [14, 15]. This validated instrument was successfully used in several studies in England as well as internationally to examine the quality of care and services received and compare them across care settings [3, 13, 16–49].

So far, little is known on the care experiences of patients in the last year of life that go beyond diagnoses and care settings in Germany. The aim of this study was to develop and validate a German version of the VOICES-SF suitable to assess the quality of care and services received across settings and healthcare providers in the context of the German health care system in the last year of life.

## Methods

### Study design and participants

Data were collected in a retrospective cross-sectional survey as part of the ‘Last Year of Life Study-Cologne (LYOLC)’ [50]. LYOLC is a mixed methods study composed of four steps (1) claims data analysis, (2) postbereavement survey with next-of-kin, (3) qualitative interviews with next-of-kin, (4) qualitative focus groups with healthcare professionals) to examine care trajectories and transitions in the last year of life until death. Sample and data collection of the survey has been described in detail elsewhere [51]. Briefly, we included relatives, friends and volunteer workers (all will be referred to as ‘informants’ hereafter) of deceased adult persons in the Cologne area. Accidental and suspicious deaths were not included. Informants had to be aged 18 years and older and proficient in German.

### Instrument development

The VOICES-SF survey is a questionnaire about experiences of end-of-life care in the last three months of life, focusing on quality of care and services received. It uses the postbereavement method to gather information from bereaved relatives, friends or carers acting as proxies. It is a survey instrument rather than a psychometric scale, questions may be added or deleted depending on a survey’s objective [14]. Using the VOICESSF (Version VOICES-SF 2014) as foundation [52], we developed a culturally adapted German version and called it ‘VOICES-Last Year of Life-Cologne (VOICES-LYOL-Cologne)’. This version evaluates care received in the last year of life, different to the original VOICESSF survey, which captures the last three months of life. This modification results from evidence showing that adding palliative care in the last 12 to 24 months of life has proven to be greatly beneficial [53, 54]. The longer period of observation enables the assessment of the introduction of palliative care services.

**Fig. 1:**
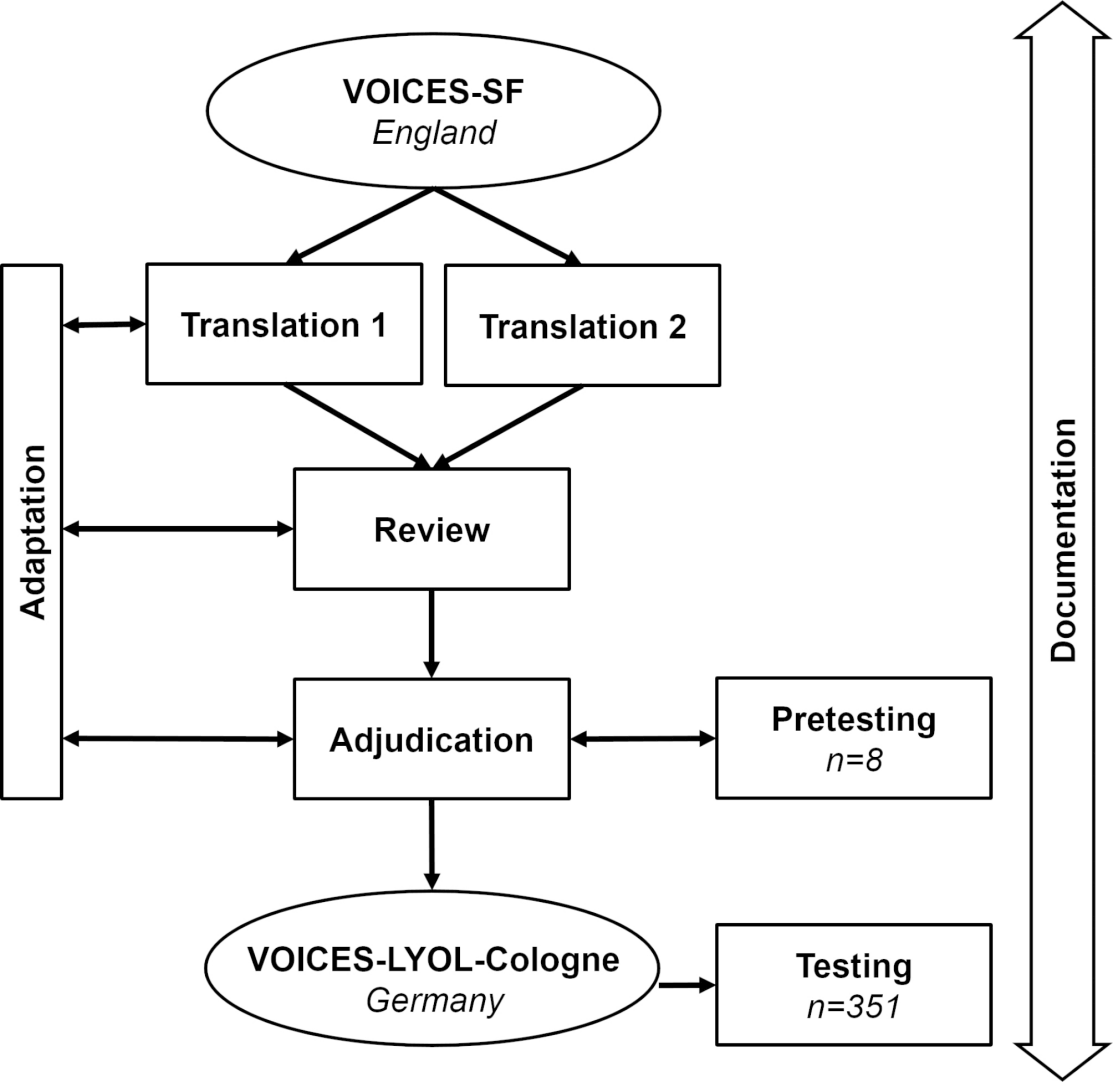
Development of the VOICES-LYOL-Cologne following the ‘TRAPD’ team approach

Figure 1 shows the translation and adaptation process. Procedures were based on the team translation approach ‘TRAPD’ (translation, review, adjudication, pre-testing, documentation) [50]. While back translation has become a controversial assessment method for questionnaire translations, ‘TRAPD’ is one of the most widely acknowledged frameworks for best practice in survey translation [51]. Two native German speakers (GD, NS) produced independent parallel translations, which were discussed with one reviewer. All of them were proficient in English. Three adjudicators (RV, JS, CR) familiar with the research project and the survey design, went through the final review version to decide on further modifications of the translation. Adaptation needs were addressed in different stages. Question adaptations were made to the content, response scales, and visual presentation of parts of the questionnaire. To produce a questionnaire that is culturally appropriate to the German health care system we conducted one group discussion with German healthcare professionals (n = 7) who work in palliative care. The resulting questionnaire was then tested in cognitive pre-tests with think-aloud technique (n = 8) to check whether (1) translated items as well as response categories were clearly understandable, (2) the questionnaire covered all important aspects of healthcare at the end of life, (3) wording and length were considered acceptable for bereaved relatives [52]. Pretesting again resulted in refinement before the adjudicators signed off on the version for final fielding. Documentation of various translation-related aspects followed the “Documenting Survey Translation” guidelines published by the - Leibniz Institute for the Social Sciences [53].

The following modifications were made:

- Revised observation period from “last three months of life” to “last year of life”.
- Added questions about the communication of the diagnosis of a life-limiting disease to assess if, by whom, when and how conversations about dying were initiated.
- Added the scale “subjective experiences of process and outcome of care in the last year of life” which comprises four key domains (relief of pain, relief of other symptoms, coordination of care, respect and dignity) in all settings of care.
- Revised terminology to align with German sample.
- Added sections related to healthcare providers additional available in the German healthcare system.
- Added single items about informal carers, place of care in the last two days of life, unsolved problems, financial problems, and demographic and clinical data.
- Harmonised response options and added “do not know” where appropriate.

The VOICES-LYOL-Cologne comprises a total of 106 items and contains skip logic so that informants only answer questions relevant to the care the patient received. In detail, it assesses care provided at home (by a nursing service, specialist palliative home care team, hospice volunteer services, general practitioner (GP), outpatient specialist physicians), care provided in a care home, hospital care (last admission), hospice care (in-patient), care provided in the last two days of life across all care settings, circumstances surrounding the death (communication of a life-limiting disease, carer support, unmet needs, financial needs, preferences and decision-making, communication of imminent death, place of death, bereavement support), and disease specific and sociodemographic data.

For each specified setting/health care provider informants could rate their subjective experiences of process and outcome of care in the last year of life on a four-point scale. Domains assessed are relief of pain, relief of other symptoms, coordination of care (1 = yes, 2 = rather yes, 3 = rather no, 4 = no), respect and dignity (1 = always, 2 = most of the time, 3 = some of the time, 4 = never). The total score is obtained by calculating the average of item scores. In addition, overall satisfaction with care in the last year of life by specified setting as well as taking all services into account is assessed on a four-point scale (1 = good, 2 = rather good, 3 = rather bad, 4 = bad). The questionnaire concludes with one free-text item to make overall comments about the care provided to the patient, which can be evaluated, for example, with qualitative content analysis [54].

#### Other instruments administered

The survey also comprised the ‘German Patient Assessment of Chronic Illness Care (PACIC) short form for bereaved persons as proxies (PACIC-S9-proxy)’ [55] in order to assess criterion validity. This validated instrument assesses patient-centred care during the last year of life retrospectively and by bereaved relatives.

### Data collection

Informants were recruited in cooperation with healthcare practitioners from Cologne between November 2017 and August 2018. Applied strategies (postal distribution, personal distribution, and self-selection) to identify potential participants have been described in detail elsewhere [56]. Study procedures were approved by the Ethics Commission of the Faculty of Medicine of the University of Cologne, Germany (#17–188).

### Data analysis

Descriptive statistics were calculated using SPSS Statistics version 26 (IBM Corp., Armonk, NY, USA). Results are presented as mean ± standard deviation (SD) and count (percentage), respectively. The presented p-values are two-sided and considered significant if p < 0.05. To assesses the validity and reliability of VOICES-LYOL-Cologne we followed the procedures applied in the validation of the modified Canadian VOICES survey [46]. This study was informed by the guidelines of the Scientific Advisory Committee of the Medical Outcomes Trust for assessing healthcare surveys [57].

#### Validity

Construct validity was investigated by generating and testing hypotheses about expected patterns of scores for groups known to differ on relevant variables (nomological network) [57, 58].

1. We expected the subjective experiences of process and outcome of care in the last year of life to be different among settings and health care providers. In previous studies informants rated hospice care more positively than homecare or hospital care [16, 18, 46, 59]. Accordingly, we hypothesised that each of the domains within hospice care would be ranked higher than care provided at home or in hospital and tested this using the Wilcoxon signed-rank test for paired samples.
2. Satisfaction with care has been found to correlate to place of death. As shown in previous VOICES studies we hypothesised that informants of people who died in hospital rated overall satisfaction with care (taking all services into account) significantly worse than in any other place of death [46, 59]. We tested this hypothesis using the Mann-Whitney U-Test.
3. The possibility of providing specialist palliative care at home was introduced in Germany in 2007 [60]. First evaluations showed that this care service enables more patients with complex symptoms and intensive care needs to die in their own home [61, 62]. We therefore expected a higher rate of home deaths in patients who received care by a specialist palliative home care team. This hypothesis was tested using the chi-square test.
4. The provision of palliative care has been found to correlate with cancer indication [63, 64]. Accordingly, we hypothesized that cancer patients received care provided by hospice, a hospital palliative care unit or by a specialist palliative home care team more often than patients with non-cancer indications and tested this using the chi-square test.

Criterion (concurrent) validity is the correlation of a scale with some other measure of the trait [65]. The PACIC-S9-proxy served as external criteria to assess criterion validity. We calculated Spearman’s rank correlation between the overall satisfaction rating on the VOICES-LYOL-Cologne and the PACIC-S9-proxy mean score, both taking all services into account. We proceeded analogously with the overall satisfaction rating by specified healthcare provider, i.e., GP, specialist physician and hospital doctor on the VOICES-LYOL-Cologne and the PACIC-S9-proxy (mean score).

#### Reliability

Cronbach’s alpha was used to assess the internal consistency of the scale “subjective experiences of process and outcome of care in the last year of life”. Cronbach’s alpha indicates whether an item of a scale is appropriate for assessing the underlying concept of its scale [66]. Commonly accepted are values above 0.70 for group comparisons [57].

As a surrogate for test-retest-reliability, we assessed whether overall satisfaction rating of care differed according to the length of time since the patient’s death (reproducibility) and used the Kruskal-Wallis test. This approach was applied by Seow et al. to evaluate the Canadian VOICES survey since retesting bereaved relatives was not feasible [46]. We expected stability of the instrument over time if informants who respond closer to the patient’s death do not differ systematically from those responding longer after a patient’s death. We defined four equally sized groups (≤ 111 days, 112 to 215 days, 216 to 331 days, ≥ 332 days) to perform this test.

## Results

### Characteristics of the sample

365 informants returned the questionnaire. The response rate depended on the recruitment strategy and differed between 10.3% for personal distribution, 21.1% for postal distribution and 74.9% for self-selection. 14 questionnaires were excluded due to a lack of inclusion criteria. The remaining 351 questionnaires were mainly answered by a spouse or a child (81.8%), the majority was female (71.5%). The decedents were 76.5 ± 13.0 years old and 52.1% were female. The main illnesses reported were cancer (59.5%) and cardiovascular diseases (40.5%). Most of the decedents (97.2%, n = 341) had received care from multiple, at least two, settings or health care providers in the last year of life. Characteristics of the deceased patients and their informants are presented in Table 1.

**Table 1:** Demographics and characteristics of deceased patients and informants (N = 351)

	n	(%)
Deceased age at death (years)		
18–29	1	(0.3)
30–49	6	(1.7)
50–64	65	(18.5)
65–79	112	(31.9)
80+	167	(47.6)
Deceased gender		
Female	183	(52.1)
Male	168	(47.9)
Deceased ethnic group		
German	340	(96.9)
Other	11	(3.1)
Deceased illnesses in the last year of life^a^		
Cancer	209	(59.5)
Cardiovascular disease	142	(40.5)
Neuro-psychological disease	115	(32.8)
Disease of the respiratory system	103	(29.3)
Kidney disease	52	(14.8)
Diabetes mellitus	45	(12.8)
Liver disease	15	(4.3)
Settings/Providers of care^b^		
Stayed in hospital (n = 350)^c^	320	(91.4)
General practitioner (n = 349)^c^	305	(87.4)
Outpatient specialist physician (n = 350)^c^	270	(77.1)
Homecare: nursing service (n = 348)^c^	157	(45.1)
Homecare: specialist palliative home care team (n = 348)^c^	135	(38.8)
Stayed in care home (n = 335)^c^	75	(22.4)
Stayed in hospice (n = 340)^c^	64	(18.8)
Homecare: hospice volunteers (n = 348)^c^	23	(6.6)
Place of death		
Hospital	148	(42.2)
At home	97	(27.6)
Hospice	61	(17.4)
Care home	41	(11.7)
Somewhere else	4	(1.1)
Informant relation to deceased		
Spouse	149	(42.5)
Son/daughter	138	(39.3)
Other relative	44	(12.5)
Friend	12	(3.4)
Other	8	(2.3)
Informant age (years)		
18–29	2	(0.6)
30–49	46	(13.1)
50–64	154	(43.9)
65–79	118	(33.6)
80+	31	(8.8)
Informant gender		
Female	251	(71.5)
Male	100	(28.5)

^a^ Multiple responses were possible

^b^ For each specific setting/healthcare provider, informants could indicate whether the decedent had at least one contact at any time during his/her last year of life (e.g. “Did your relative stay in hospital at any time during the last year of life?”, “Did your relative receive care by a GP in the last year of life?”)

^c^ Due to missing data, the individual n are indicated

See also Schippel et al. [67] and Voltz et al. [56]

### Validity

#### Construct validity

1. Subjective experiences of process and outcome of care in the last year of life differed between health care providers. As expected, hospice care was rated best in every single domain (Table 2). Significant differences between ratings of hospice care and homecare were found for relief of pain (Z = 2.105, p = 0.035, n = 45), relief of other symptoms (Z = 3.273, p = 0.001, n = 47), coordination of care (Z = 3.214, p = 0.001, n = 41), respect and dignity (Z = 3.243, p = 0.001, n = 59). Between hospice care and hospital care significant differences were found for relief of pain (Z = 3.622, p < 0.001, n = 39), relief of other symptoms (Z = 2.586, p = 0.010, n = 34), coordination of care (Z = 2.759, p = 0.006, n = 22), respect and dignity (Z = 3.968, p < 0.001, n = 39).
2. Informants’ overall satisfaction with care in the last year of life varied depending on the place of death (Table 3). Dying in hospital corresponded to a lower overall satisfaction rating (mean = 2.08 (SD = 0.84), 26.2% rated care as “rather bad” or “bad”) compared to dying in any other place (mean = 1.6 (0.63), 5.3% rated care as “rather bad” or “bad”)). Differences were statistically significant (U = 5892.5, p = < 0.001, n = 330).
3. As hypothesized the integration of a specialist palliative home care team was associated with the place of death (Table 4). While half of the patients treated by specialist palliative home care died at home, this was only fulfilled for 14% of patients who did not receive this service. The results show that not including a specialist palliative home care team is associated with more people dying elsewhere than at home. Differences were statistically significant (χ^2^ = 49.056, df = 1, p < 0.001, n = 345).
4. As hypothesized palliative care was more often provided to cancer patients than to other indications. The majority of cancer patients (86%) received care either provided by hospice, a hospital palliative care unit or a specialist palliative home care team (Table 5). On the other hand, only one third of patients with non-cancer indications received specialist palliative care. Differences were statistically significant (χ^2^ = 88.221, df = 1, p < 0.001, n = 320).

**Table 2:** Subjective experiences of process and outcome of care in the last year of life

	Hospice (in-patient)		Homecare		Hospital care (last admission)		Hospice vs. Homecare	Hospice vs. Hospital
**Domains**	n	Mean (SD)	n	Mean (SD)	n	Mean (SD)	p-value	p-value
Relief of pain^a^	60	1.23 (0.50)	246	1.76 (0.84)	188	1.75 (0.80)	0.035	< 0.001
Relief of other symptoms^a^	58	1.41 (0.68)	255	2.00 (0.89)	180	1.95 (0.83)	0.001	0.010
Coordination of care^a^	50	1.18 (0.63)	244	1.73 (0.89)	133	2.63 (1.18)	0.001	0.006
Respect and dignity^b^	64	1.09 (0.34)	320	1.25 (0.41)	201	1.74 (0.76)	0.001	< 0.001

^a^ Answer category ranging from “yes” (= 1) to “no” (= 4)

^b^ Answer category ranging from “always” (= 1) to “never” (= 4)

**Table 3:** Overall satisfaction with care in the last year of life by place of death

	‘Overall, and taking all services into account, how would you rate his/her care in the last year of life?’								
**Place of death**	**Good**		**Rather good**		**Rather bad**		**Bad**		
	n	(%)	n	(%)	n	(%)	n	(%)	Mean (SD)
Hospital (n = 65)	16	(24.6)	32	(49.2)	13	(20)	4	(6.2)	2.08 (0.84)
Non-hospital (n = 265)	122	(46)	129	(48.7)	11	(4.2)	3	(1.1)	1.6 (0.63)

Mann-Whitney U-Test: U = 5892.5, p = < 0.001, n = 330

**Table 4:** Association of specialist palliative home care and place of death

		Place of death			
		Home (n = 97)		Non-home (n = 248)	
		n	(%)	n	(%)
**Patient received care from a specialist palliative home care team**	Yes (n = 135)	67	49.6	68	50.4
	No (n = 210)	30	14.3	180	85.7

Chi-square test: χ^2^ = 49.056, df = 1, p < 0.001, n = 345

**Table 5:** Association of cancer indication and the provision of specialist palliative care

		Patient received specialist palliative care^a^	
		Yes (n = 213)	No (n = 107)
**Indication**	Cancer (n = 200)	172 (86%)	28 (14%)
	Non-cancer (n = 120)	41 (34.2%)	79 (65.9%)

^a^ Care may be provided by in-patient hospice, a hospital palliative care unit or by a specialist palliative home care team

Chi-square test: χ^2^ = 88.221, df = 1, p < 0.001, n = 320

#### Criterion validity

Ratings of overall satisfaction on the VOICES-LYOL-Cologne were found to highly correlate with the mean scores of PACIC-S9 Proxy. A significant correlation was found for taking all services into account (r_s_ = 0.400, p < 0.001, n = 225) as well as for specified healthcare providers: care by a GP (r_s_ = 0.522, p < 0.001, n = 93), care by a specialist physician (r_s_ = 0.491, p < 0.001, n = 69), care by a hospital doctor (r_s_ = 0.481, p = 0.001, n = 43).

### Reliability

#### Internal consistency

Cronbach’s alpha was calculated for each given setting (hospital: α = 0.829, care home: α = 0.821, hospice: α = 0.814, home: α = 0.652).

#### Reproducibility

Regarding overall satisfaction with care, there was no statistical difference between relatives who responded closer to the patient’s death and those responding longer after a patient’s death (n = 325, Kruskal-Wallis H = 1.59, p = 0.662).

## Discussion

This study describes the development of the VOICES-LYOL-Cologne – an adapted German version of the English VOICES-SF – and evaluates its validity and reliability. We used the team translation approach ‘TRAPD’ and included healthcare professionals as experts to produce a questionnaire that is culturally appropriate to the German health care system. The evaluation followed procedures applied in the validation study of the Canadian VOICES survey including a multitude of statistical tests [46]. Hypotheses about expected patterns of scores for groups known to differ on relevant variables were tested and generated results that corresponded with expectations. Criterion validity was also evident against the PACIC-S9 Proxy, an instrument to evaluate the level of patient-centeredness in the care during the last year of life [55]. Good internal consistency was found for the scale “subjective experiences of process and outcome of care in the last year of life” for each given care setting, except for the homecare setting. Test-retest analyses revealed no significant differences in satisfaction ratings according to the length of time since the patient’s death. Overall, our data demonstrate the feasibility of collecting patient care experiences reported by proxy-respondents across multiple care settings.

### Comparison with previous research

VOICES’ content is based on patients’, bereaved relatives’ and healthcare professionals’ views about what is important at the end of life and has been developed in England [14]. Further versions are already available and have been published for the use in Sweden, Denmark, New Zealand, Canada, Thailand, Italy, and a Bengali-speaking community in London [19, 30, 31, 39, 44, 46, 68]. This study used the most recent version of the VOICES-SF from 2014 [69] as foundation to develop a culturally adapted German version. The adaptation process comprised alterations independent of unavoidable translation change. The main objective was to render questions culturally and linguistically appropriate. Since adapted questions should be treated as new questions, we do not recommend to compare questions of the VOICES-LYOL-Cologne with the original VOICES-SF or the other translations and their performance.

VOICES-LYOL-Cologne evaluates care received in the last year of life and incorporates the communication of the diagnosis of a life-limiting disease, different to the original VOICESSF survey, which captures the last three months of life. This modification results from evidence showing that adding palliative care in the last 12 to 24 months of life has proven to be greatly beneficial [70, 71]. Therefore, while evaluating experiences of care at the end of life, consideration could now also be given to the introduction of these services [51]. Furthermore, key domains of the subjective experiences of process and outcome of care in the last year of life with individual health care providers were added. This scale enables a more detailed assessment and comparison of care experiences across care settings.

### Strengths and limitations

Experience measures are used to capture what really matters to patients to identify gaps in care and to make service improvements [11]. The instruments available so far focus on single care settings, are restricted to the dying phase or examine care in case of a concrete diagnosis. The VOICES-LYOL-Cologne assesses care in the entire last year of life. It proceeds irrespective of the underlying diagnosis and includes all healthcare providers involved in patients’ care. This allows us to gain a broad overview of care processes in the last year of life, to analyse potential correlations, and to inform quality improvements.

We conducted a retrospective survey with bereaved relatives as proxies for deceased patients – as intended by the original VOICES survey [13]. Postbereavement studies may be influenced by participant’s memory, feelings or the level of agreement with patient’s views [72, 73]. Our data did not show any significant differences in overall satisfaction with care regarding time since patient’s death to survey completion. Evidence shows that salient events may be recalled more accurately and that the level of agreement may be good on service evaluations and observable symptoms [74–76].

This study was part of the mixed methods ‘Last Year of Life Study-Cologne (LYOLC)’ conducted in the city of Cologne, Germany [50]. Data of the postbereavment survey were based on a purposive sample since a population-based survey was not feasible. Patients from palliative care services were overrepresented, which can be attributed to the recruitment strategy. Nevertheless, the sample was in line with the age and gender distribution of people who died in the city of Cologne [77, 78].

## Conclusion

VOICES-LYOL-Cologne is the first German instrument to analyse care experiences in the last year of life in a comprehensive manner. The reliability of one core scale to assess “subjective experiences of process and outcome of care in the last year of life” was tested and showed satisfactory psychometric properties. In addition to recording the provision of services and circumstances surrounding death, VOICES-LYOL-Cologne might enable the comparison of care quality between settings. The instrument will be available on request and encourages further research in German-speaking countries. Future studies should use the survey in broader, more representative populations. VOICES-LYOL-Cologne may also be used to benchmark individual providers, who may benefit from feedback and to inform local as well as national quality improvement activities. In England the VOICES-SF survey was used annually as part of the “National Survey of Bereaved People” to achieve the goals of the “End of Life Care Strategy” [79]. VOICES-LYOL-Cologne may also be used to improve regional care in the last year of life. In Cologne for example, a PDSA cycle has been set up by analysing patient care experiences [56]. A working group to improve regional care and further research projects have already resulted [80, 81]. To gain in-depth insight into the reasons for transitions and the effects on the quality of life of the patients and their relatives, future studies may use a mixed-methods design as we did in the project LYOL-C [82]. Qualitative interviews with bereaved informal caregivers after completing the questionnaire may gain in-depth insight into patient trajectories [67]. Furthermore, the views of healthcare professionals may be important to analyse challenges associated with transitions in the last year of life and identify possibilities for improvements [83].

## Data Availability

The datasets used and/or analysed during the current study are available from the corresponding author upon reasonable request and following application to CoRe-Net (Core-Net@uk-koeln.de).

## Abbreviations

CoRe-Net: Cologne Research and Development Network
GP: general practitioner
PACIC-S9-proxy: Patient Assessment of Chronic Illness Care- short form containing nine items for completion by proxy informants
PREM: patient reported experience measures
TRAPD: translation, review, adjudication, pre-testing, documentation
VOICES: Views of Informal Carers’ Evaluation of Services
VOICES-LYOL-Cologne: Views of Informal Carers’ Evaluation of Services-Last Year of Life-Cologne
